# An analysis of variations in the bronchovascular pattern of the right upper lobe using three-dimensional CT angiography and bronchography

**DOI:** 10.1007/s11748-015-0531-1

**Published:** 2015-02-28

**Authors:** Toshiteru Nagashima, Kimihiro Shimizu, Yoichi Ohtaki, Kai Obayashi, Seiichi Kakegawa, Seshiru Nakazawa, Mitsuhiro Kamiyoshihara, Hitoshi Igai, Izumi Takeyoshi

**Affiliations:** 1 Department of Thoracic and Visceral Organ Surgery, Gunma University Graduate School of Medicine, 3-39-22 Showa-machi, Maebashi, Gunma 371-8511 Japan; 2 Department of General Thoracic Surgery, Maebashi Red Cross Hospital, Maebashi, Japan

**Keywords:** Lung anatomy, Three-dimensional CT, Lung cancer surgery, Segmentectomy

## Abstract

**Objectives:**

General thoracic surgeons must be familiar with anatomical variations in the pulmonary bronchi and vessels. We analyzed variations in the bronchovascular pattern of the right upper lung lobe using three-dimensional CT angiography and bronchography and then compared our results with those of previous reports.

**Methods:**

We reviewed anatomical variations in the right upper pulmonary bronchus and vessels of 263 patients using 3DCT angiography and bronchography images obtained using a 64-channel multidetector CT and workstation running volume-rendering reconstruction software.

**Results:**

Variations in the pulmonary vein were classified into four types: the “anterior-plus-central vein type” was the most common, noted in 219 cases (83.2 %). The “anterior vein type” was evident in 23 cases (8.8 %), a significantly lower incidence than in previous reports (*p* < 0.001). Also, the branching patterns of the segmental arteries of the pulmonary artery differed partially from those noted in previous reports. Furthermore, we identified some new variations. The “B^1^- or B^2^-defective branch type” bronchus was noted in 19 cases (7.2 %), which was a higher prevalence than that in previous reports.

**Conclusion:**

We explored the bronchovascular pattern and the frequency of variations in the right upper lobe using a large number of 3DCT images. The incidences of variations differed, sometimes significantly, from those noted by previous reports. Moreover, we report some new branching variations. Our data can be used by thoracic surgeons to perform safe and precise lung resections.

**Electronic supplementary material:**

The online version of this article (doi:10.1007/s11748-015-0531-1) contains supplementary material, which is available to authorized users.

## Introduction

Lung screening using computed tomography (CT) has recently become widespread, and many small lung lesions are detected using this method. In particular, the incidence of ever-smaller non-small cell lung carcinoma (NSCLC), which may have more indolent biological behavior, such as bronchioloalveolar carcinoma, is increasing [1–3]. Several authors have reported that the prognosis of selected patients undergoing sublobar resection is not inferior to lobectomy with small-sized NSCLC [4–6]. Therefore, the requirement for anatomical pulmonary segmentectomy, which preserves more lung function compared to lobectomy, has also increased. However, a segmentectomy is technically more difficult than a standard lobectomy because of the anatomical complexity of the lung, featuring both vascular and bronchial structures that vary at different levels. Therefore, complete knowledge of the pulmonary bronchovascular pattern including rare anatomical variations and a full understanding of individual anatomy by general thoracic surgeons has become more important for performance of safe and precise pulmonary surgery.

Recently, advances in multidetector row computed tomography (MDCT) and imaging techniques using volume-rendering reconstruction software have allowed reconstruction of three-dimensional (3D) images [7]; a high detection rate of pulmonary vessels using the 3D images has been reported [8, 9]. In addition, the efficacy of 3DCT images for preoperative assessment of thoracic surgery has also been described [10].

Although 3DCT images are useful in understanding individual anatomy, 3DCT imaging is not always available. Moreover, even if we obtain 3DCT images preoperatively, thoracic anatomy is difficult to interpret using 3DCT without underlying data from anatomical atlases and texts. However, currently available data on pulmonary bronchovascular patterns are limited to a few cadaveric studies by Boyden et al. [11] and Yamashita [12] between the 1950s and 1980s.

The purpose of this study was to analyze variations in the pulmonary bronchovascular pattern using 3DCT angiography and bronchography (3DCTAB) and to construct the underlying data of pulmonary segmental structures to replace previous cadaveric studies [11, 12] and for use in anatomical atlases and texts of thoracic surgery. We selected the right upper lobe (RUL) for this first report, because it undergoes more lobectomies and segmentectomies compared with other lobes [13, 14]. Analyses of the bronchovascular pattern and its frequencies are ongoing in the right middle lobe, right lower lobe, left upper, and lower lobes.

## Patients and methods

### Reconstruction of 3DCTAB imaging

Bronchovascular patterns revealed by 3DCTAB imaging were analyzed using a 64-channel MDCT (SOMATOM Definition Flash; Siemens Healthcare, Berlin, Germany). A total of 35 ml of contrast agent was mechanically injected at 5 ml/s, followed immediately by injection of 20 ml of saline. A solid image was constructed from 1.0-mm data slices of contrast-enhanced CT images with the aid of 3D volume rendering. The volume data from both arterial and venous phases were transferred to a workstation running volume-rendering reconstruction software (Ziostation2; Ziosoft, Tokyo, Japan) that converted the data to the 3DCT angiographic format. The 3D reconstruction of the bronchial tree involved mathematical morphology-based 2D segmentation of axial images, followed by restoration via manual addition of segments from 2D axial images to form 3D images. Radiology technicians processed all 3D images and respiratory surgeons confirmed the validity of the reconstructions.

### Patient preparation and examination

Between January 2010 and January 2014, 263 patients with respiratory or mediastinal lesions underwent 3DCTAB prior to surgery. Three of the 263 cases (1.1 %) were excluded, because the subsegmental branches of the pulmonary vessels were not adequately represented on 3DCTAB. Thus, the 260 most recent consecutive cases were analyzed for variations in the pulmonary vessel pattern. The patient characteristics are summarized in e-Table 1. Forty-six of the 263 cases (17 %) yielded insufficient data for a bronchial pattern analysis because the segmental bronchi were not completely visible on 3DCT bronchography. Thus, 214 cases were analyzed for variations in the pulmonary bronchial pattern.

The frequencies of each bronchovascular pattern in our study and those of previous studies (Yamashita [12] and Boyden [11]) were compared using the *χ* ^2^ test. However, some parts of the branching patterns have not been described in previous studies [11, 12], and we could not compare our data with these previous data. All statistical analyses were performed using SPSS statistics ver. 22 software (SPSS Inc., Chicago, IL, USA). This retrospective study was approved by the Research Ethics Committee at Gunma University Hospital.

### Detection rate of 3DCTAB

To assess the detection rate of pulmonary vessels of 3DCTAB, the findings were retrospectively compared with actual anatomy using operation reports and intraoperative videos. A total of 155 patients who underwent right upper lobectomy, right middle lobectomy, or left upper lobectomy were included in this verification study, because the discrepancy between the 3DCTAB findings and anatomical variations in the pulmonary artery could only be compared for these surgical procedures.

### Definition of pulmonary artery and vein

**Fig. 1:**
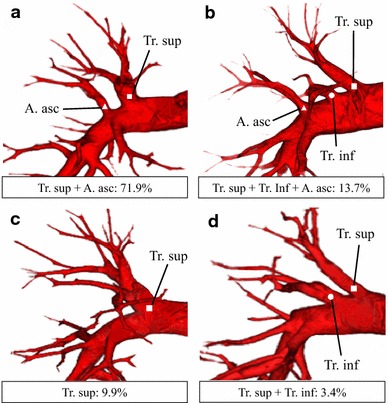
Types of branching in the right upper lobe arteries. **a** Tr. sup + A. asc type. **b** Tr. sup + Tr. inf + A. asc type. **c** Tr. sup type. **d** Tr. sup + Tr. inf type. *Squares* trunks superior, *circles* trunks inferior, *triangles* ascending artery

Branching of the pulmonary artery was defined as four names as follows: trunk superior (Tr. sup): Tr. sup is the first branch of the right main pulmonary artery; trunk inferior (Tr. inf): Tr. inf is the second branch of the right main pulmonary artery and arises from the mediastinal portion of the artery, between the distal region of Tr. sup and the proximal region of the first middle lobe of the pulmonary artery; ascending artery (A. asc): A. asc arises from the interlobar portion of the right pulmonary artery and generally branches from the distal region of the first middle lobe artery; recurrent artery (A. rec): A. rec is the artery that branches from the Tr. sup and crosses above the right upper lobe bronchus to S^2^ (S. dorsal) (Fig. 1a–d).

**Fig. 2:**
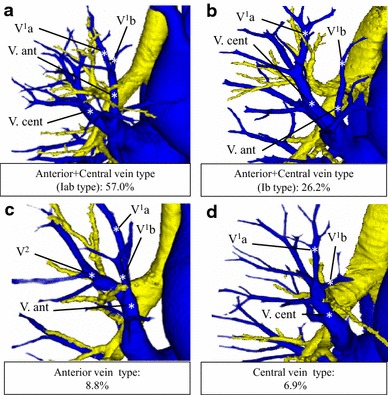
Types of branching in the right upper lobe veins. **a** Anterior with central vein type (Iab type). **b** Anterior with central vein type (Ib type). **c** Anterior vein type. **d** Central vein type

Branching of the pulmonary vein was defined as two names as follows: anterior vein (V. ant): V. ant is the vein that arises from the mediastinal side and ascends the anterior face of the upper lobe bronchus; central vein (V. cent): V. cent is the vein that arises from the interlobar side and ascends through the center of the lung, between B^2^ and B^3^ (Fig. 2a–d).

## Results

### Detection rate of 3DCTAB

A total of 455 pulmonary arterial branches were resected using the above lung resection procedures. All of the resected pulmonary arterial branches in 149 patients (96.1 %) were detected by 3DCTAB. Only six pulmonary arteries (1.3 %) of the 455 resected branches were not detected by 3DCTAB, including two right ascending arteries, two right middle lobe arteries, one left lingular artery, and one left A^1+2^c, which were all <1.5 mm in diameter. Thus, the detection rate of our 3DCTAB (98.7 %) was feasible for this study.

### Pulmonary artery

Pulmonary artery branching patterns were classified into four types (Table 1; Fig. 1a–d). The “Tr. sup + A. asc type” was evident in 189 cases (71.9 %) (Fig. 1a) and was the most common type. Tr. inf was seen in 45 cases (17.1 %), and these cases were further divided into two types. The “Tr. sup + Tr. inf + A. asc type” was evident in 36 cases (13.7 %) (Fig. 1b) and the “Tr. sup + Tr. inf type” was seen in nine cases (3.4 %) (Fig. 1d). Only the Tr. sup branched from the main pulmonary artery in the “Tr. sup type” and no other branches were evident. This variation was seen in 26 cases (9.9 %) (Fig. 1c). In 225 examples of A. asc, 28 had two ascending arteries (12.4 %). Our data did not differ significantly from those of Yamashita [12].

**Table 1:** Branching types of the right upper lobe artery, vein, and bronchus

	Our study (*n* = 263)		Yamashita (*n* = 170)		*p* value	Figures
	No.	%	No.	%		
Pulmonary artery						
Tr. sup + A. asc type	189	71.9	133	78.0	0.13	1a
Tr. sup + Tr. inf + A. asc type	36	13.7	22	13.1	0.82	1b
Tr. sup type	26	9.9	10	5.9	0.14	1c
Tr. sup + Tr. inf type	9	3.4	5	3.0	0.78	1d
N/A	3	1.1	–	–	–	–
Pulmonary vein						
Anterior with central vein type (Iab type)	150	57.0	86	50.8	0.18	2a
Anterior with central vein type (Ib type)	69	26.2	33	19.3	0.10	2b
Anterior vein type	23	8.8	37	21.7	<0.001	2c
Central vein type	18	6.9	14	8.2	0.58	2d
N/A	3	1.1	–	–	–	–
Bronchus						
Bifurcated type	77	29.3	90	53	<0.001	
B^1^ + B^2^, B^3^	38	14.4	46	27	0.001	3b
B^1^ + B^3^, B^2^	23	8.8	19	11	0.40	3c
B^2^ + B^3^, B^1^	16	6.1	25	15	0.003	3d
Defective B^1^ or B^2^	19	7.2	7	4	0.18	
B^2^ + BX^1^a, B^3^ + BX^1^b	13	4.9	NR	–	–	3e
B^1^ + BX^2^a, B^3^ + BX^2^b	6	2.3	7	4	0.27	
Trifurcated type (B^1^, B^2^, B^3^)	116	44.1	51	30	0.003	3a
Quadrivial type	2	0.8	22	13	<0.001	3f
N/A	49	18.6	–	–	–	–

*N/A* not available, *NR* the type was not referred

### Segmental arteries

#### The apical segmental artery (A^1^)

The A^1^a and A^1^b branching patterns were divided into two types (e-Table 2; e-Fig. 1a, b). In 225 cases (85.6 %), the A^1^a + A^1^b branched together directly from the Tr. sup (e-Fig. 1a). However, in 35 cases (13.3 %), only A^1^a branched directly from the Tr. sup, and A^1^b branched from an A^3^ that bifurcated from the Tr. sup (e-Fig. 1b). The former pattern occurred significantly more frequently than noted in previous studies (Yamashita, *p* < 0.001; Boyden, *p* = 0.01) [11, 12].

#### The posterior segmental artery (A^2^)

Normally, A^2^ consists of A. rec and A. asc. The A^2^a and A^2^b branching patterns were classified into three types (e-Table 2; e-Fig. 2a–c). The “A^2^a branching from A. rec, A^2^b branching from A. asc” type was noted in 122 cases (46.4 %) (e-Fig. 2a) and was the most common type. We did not encounter the “A^2^a branching from A. asc, A^2^b branching from Tr. inf” type (as noted in 1.2 % of the cases of Yamashita, cited above) [12]. Some new variations in the branching pattern were identified. A^2^ (A^2^b alone, or both the A^2^a and A^2^b branches) arose with A^3^, in a common trunk, from the Tr. sup or Tr. inf. This new variation was present in 11 cases (4.2 %) (e-Fig. 2d). A^2^ (A^2^b alone or both the A^2^a and A^2^b) branching with A^6^, as a common trunk from the intermediate pulmonary artery, was evident in 13 cases (5.0 %) (e-Fig. 2e, f).

#### The anterior segmental artery (A^3^)

The A^3^ branching pattern was classified into five types (e-Table 2; e-Fig. 3a–e). The “A^3^a + A^3^b from Tr. sup” type was noted in 180 cases (68.5 %) (e-Fig. 3a) and was the most common type observed. The “A^3^a branching from A. asc, A^3^b branching from Tr. inf” type was noted in only five cases (1.9 %) (e-Fig. 3e). A new anomalous A^3^ branching pattern was identified in one case (0.4 %); A^3^a branched from the right middle lobe artery (e-Fig. 3f). The frequencies of variations differed, sometimes significantly, from those noted by Yamashita and Boyden [11, 12].

### The pulmonary vein

The pulmonary vein branching pattern was classified into four types (Table 1; Fig. 2a–d). An “anterior with central vein type” was evident in 219 instances (83.2 %) (Fig. 2a, b) and was further subclassified into two types. In the Iab type, V^1^a and V^1^b drained into V. ant, and this variation was present in 150 cases (57.0 %) (Fig. 2a). In the Ib type, only V^1^b drained into V. ant, whereas V^1^a drained into V. cent. This variation was seen in 69 cases (26.2 %) (Fig. 2b). The “anterior vein type”, in which V^1−3^ mainly drained into V. ant, was evident in only 23 cases (8.8 %) (Fig. 2c). This type was seen significantly less frequently than in previous reports (Yamashita, *p* < 0.001) [12]. The “central vein type”, in which V^1−3^ drained into V. cent, was seen in 18 cases (6.9 %) (Fig. 2d). An anomalous V^2^ drainage pattern, termed “Aberrant V^2^”, was also identified; V^2^ drained into the inferior pulmonary vein, crossing behind the intermediate bronchus. This relatively infrequent type was present in five cases (1.9 %) (e-Fig. 4).

### Bronchus

**Fig. 3:**
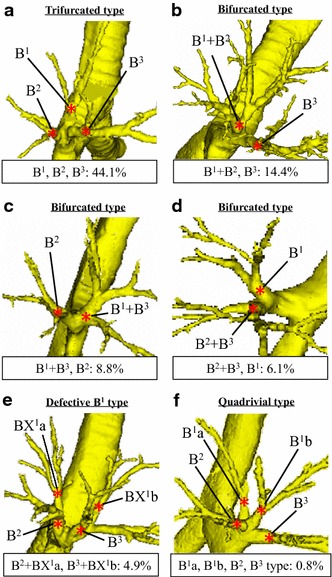
Types of branching of the right upper lobe bronchus. **a** The trifurcated type (B^1^, B^2^, and B^3^). **b** The B^1+2^, B^3^ type. **c** The B^1+3^, B^2^ type. **d** The B^2+3^, B^1^ type. **e** The defective B^1^ type (B^2^ + BX^1^a and B^3^ + BX^1^b). **f** The quadrivial type (B^1^a, B^1^b, B^2^ and B^3^)

Branching of the right upper bronchus was classified into three types (Table 1; Fig. 3a–f). The trifurcated type (the “B^1^, B^2^, and B^3^” type) was evident in 116 cases (44.1 %; Fig. 3a), and was the most common. The bifurcated type was seen in 77 cases (29.3 %) (Fig. 3b–d). This type was further divided into three subtypes. The “B^3^ and B^1+2^” type was evident in 38 of 263 cases (14.4 %) (Fig. 3b) and was the most common. B^1^ or B^2^ was rarely absent. In such instances, S^1^ or S^2^ was supplied by an accessory bronchus from B^2^ or B^3^. The “defective B^1^” type (B^2^ + BX^1^a and B^3^ + BX^1^b) was seen in 13 cases (4.9 %) (Fig. 3e), and the “defective B^2^” type (B^1^ + BX^2^a and B^3^ + BX^2^b) was evident in 6 cases (2.3 %). The quadrivial type was seen in only two cases (0.8 %) and was significantly less frequent than in previous reports (Yamashita, *p* < 0.001; Boyden, *p* < 0.001) (Fig. 3f) [11, 12]. The frequency of the trifurcated type was higher (*p* = 0.003) and that of the bifurcated type lower than those noted by Yamashita (*p* < 0.001), but they did not differ significantly from those reported by Boyden [11, 12].

## Discussion

For thoracic surgeons, knowledge of pulmonary bronchovascular patterns including rare anatomical variations is extremely important to perform safe and accurate pulmonary resection. We report here a systematic radiological analysis of the pulmonary structure of the RUL and the discrepancy between our results and those of previous reports [11, 12]. A few reports have focused on thoracic anatomical abnormalities detected with the aid of 3DCT [15, 16]. However, no report has systematically explored the bronchovascular pattern with the aid of 3DCT imagery. This is therefore the first radiological systematic analysis of pulmonary structure and, moreover, our study included the greatest number of cases (*n* = 263) of all relevant reports (Yamashita, *n* = 170; Boyden, *n* = 50).

The branching types noted in the right pulmonary artery were similar to those in previous reports [11, 12]. However, some previous definitions of the pulmonary artery were ambiguous, so these were redefined in this work. Yamashita defined the Tr. inf as the inferior branch of the Tr. sup when the Tr. sup is bifurcated [12]. However, in reality, Tr. inf does not “bifurcate” from Tr. sup, but rather branches peripherally from the main trunk. Furthermore, although the Tr. inf is usually described as branching from the mediastinal side, and A. asc branches interlobarly, the borderline between the mediastinum and the interlobar plane is ambiguous. Thus, Tr. inf and A. asc are often confused. We newly defined that the boundary of the Tr. inf and A. asc was the first middle lobe artery. We encountered cases in which the middle lobe artery branched more peripherally than usual, but this was rare (7/225 cases; 3.1 %) and did not affect the overall results, even compared to previous reports. Thus, our new definition appears to be acceptable.

In contrast, in the right pulmonary vein, the frequency of each branching type differed greatly from that of previous reports. In our study, the “anterior vein type” was seen in only 8.8 % of cases, as compared to 21.7 % in a previous study. This is the most prominent difference between our data and those of previous reports. In a previous report, the combined V. cent frequency was 77 % [12], but it was 90 % in our study. We suggest that this reflects the differences between anatomical and radiological evaluation. It is difficult to clearly distinguish V. cent, which runs between B^2^ and B^3^, and V^2^t, which runs under B^2^ and B^3^, using anatomical techniques. 3DCTAB is considerably more effective in this regard.

Our data on the right upper bronchus also differ from those of previous reports [11, 12]. In the present study, the most common type was the “trifurcated”, evident in 44.1 % of cases (Yamashita 30 %, Boyden 30 %; *p* = 0.003) [11, 12]. The quadrivial type was seen in only two cases (0.8 %) and thus was significantly less common than in previous reports (Yamashita 13 %, Boyden 16 %; *p* < 0.001) [11, 12]. Furthermore, we found that the defective B^1^ and B^2^ patterns were relatively common. If bronchial patterns are analyzed anatomically, the lung parenchyma and pulmonary vessels complicate studying bronchial branching. However, 3DCTAB can simply erase images of the parenchyma and vessels, allowing bronchial branching to be examined from all perspectives. We suggest that our data are more accurate than those of previous reports [11, 12].

These anatomical data, along with all possible variations, should be noted preoperatively. When the separation of the interlobular fissure is incomplete, preoperative knowledge of variations revealed in our study, such as “Aberrant V^2^” and the “A^2^ with the A^6^ type”, is necessary to safely create an interlobular fissure between the upper and lower lobes.

During anatomical sublobar resection, understanding the branching types of the pulmonary vein is extremely important, because the segmental veins bind the pulmonary segments, and the surgical approach varies according to its branching type. For example, V^1^b, which is an intersegmental vein between S^1^ and S^3^, usually drains into the V. ant. However, V^1^b drains into V. cent only in the “central vein type”. Therefore, a different approach is required to perform anatomical intersegmental dissection between S^1^ and S^3^ in this type. Thus, preoperative simulation of individual bronchovascular patterns using MDCT or 3DCT images, as well as knowledge of rare branching patterns of segmental bronchi and arteries, is necessary to perform precise sublobar resection.

Although 3DCT images may be a breakthrough tool in preoperative simulation of lung resection, 3DCT imaging is not available for all patients. Furthermore, in our experience, even if an actual preoperative 3DCT image is obtained, it is difficult to understand the thoracic anatomy without knowledge of the pulmonary bronchovascular patterns and their incidences. Thus, our data of bronchovascular patterns including rare anatomical variations using the accumulated 3DCTAB images of 263 patients in our institute may facilitate safe and accurate lung resection by general thoracic surgeons, regardless of the presence or absence of preoperative 3DCT images.

There were several limitations in this study. First, we could not obtain adequate images of segmental vessels and bronchi from some patients. Although the number of insufficient cases was small, they may have biased our results. Second, our study was an anatomical analysis based on 3DCT findings; thus, it is possible that there were differences in our data from actual anatomy. The detection rate of our 3DCTAB was 98.7 %, so we thought it was reasonable to compare our 3DCTAB data with those of previous reports based on actual anatomy. However, the limitation of differences from actual anatomy still remains, because we did not compare our data with that of the resected lung.

## Conclusion

This is the first report on variations in the bronchovascular patterns of the RUL with descriptions of rare branching patterns. We extracted data from a large number of 3DCTAB images, which aided in understanding individual variations in thoracic anatomy. Our data were collected with maximum use of 3DCTAB and should be referred to by thoracic surgeons prior to performing lung resection, particularly lobectomy and segmentectomy.

## Electronic supplementary material

Below is the link to the electronic supplementary material.

**e-Fig.** **1.** (a) – (b) Branching patterns of the apical segmental arteries (A^1^). (a) A^1^a and A^1^b branching directly from Tr. sup. (b) A^1^a branching from Tr. sup, and A^1^b branching from A^3^. □:Trunks superior △:Ascending artery.

**e-Fig.** **2.** (a) – (f) Branching patterns of the posterior segmental arteries (A^2^). (a) A^2^a branching from Tr. sup and A^2^b branching from A. asc. (b) A^2^a and A^2^b branching together from A. asc. (c) A^2^a and A^2^b branching together from A. rec. (d) A^2^b alone, or A^2^b and A^2^a, branching from A^3^. (e) A^2^b branching from A^6^ and A^2^a branching from A.rec. (f) A^2^a and A^2^b branching from A^6^. □:Trunks superior ○:Trunks inferior △:Ascending artery

**e-Fig.** **3.** (a) – (f) Branching patterns of the anterior segmental arteries (A^3^). (a) A^3^a and A^3^b branching together from Tr. sup. (b) A^3^b branching from Tr. sup and A^3^a branching from A. asc. (c) A^3^a and A^3^b branching together from Tr. inf. (d) A^3^a branching from Tr. sup and A^3^b branching from Tr. inf. (e) A^3^b branching from Tr. inf and A^3^a branching from A. asc. (f) A^3^a branching from the middle lobe artery and A^3^b branching from Tr. sup. □:Trunks superior ○:Trunks inferior △:Ascending artery

**e-Fig.** **4.** The anomalous V^2^ drainage pattern, termed “Aberrant V^2^” (white arrow). PVI = pulmonary vein inferior

Supplementary material 1 (DOCX 20 kb)

Supplementary material 2 (PPTX 3675 kb)
