# Supplementary material for: An analysis of variations in the bronchovascular pattern of the right upper lobe using three-dimensional CT angiography and bronchography
Source: Gen Thorac Cardiovasc Surg. 2015 Feb 28;63(6):354–60. doi: 10.1007/s11748-015-0531-1 (PMC4454828; doi:10.1007/s11748-015-0531-1)
Supplement: Supplementary file 1 — Supplementary material 1 (DOCX 20 kb) [file 11748_2015_531_MOESM1_ESM.docx]

e-Table 1. Summary of patients’ characteristics.

|  | **Cases (n=263)** | |
| --- | --- | --- |
|  | **No.** | **(%)** |
| Age (year) |  |  |
| Median (range) | 66 (15-90) |  |
| Gender |  |  |
| Male | 155 | (59) |
| Female | 108 | (41) |
| Disease |  |  |
| Primary lung cancer | 176 | (67) |
| Mediastinal tumor | 33 | (13) |
| Metastatic lung cancer | 22 | (8) |
| Others | 32 | (12) |

e-Table 2. Branching patterns of the segmental arteries in the right upper lobe.

| **A^1^** | **Our study (n=263)** | | **Yamashita (n=170)** | | ***p* Value** | **Boyden (n=50)** | | ***p* Value** | **Fig.** |
| --- | --- | --- | --- | --- | --- | --- | --- | --- | --- |
|  | **No.** | **%** | **No.** | **%** |  | **No.** | **%** |  |  |
| A^1^a + A^1^b from Tr. Sup | 225 | 85.6 | 116 | 68 | < 0.001 | 36 | 72 | 0.01 | e-1a |
| A^1^a from Tr. Sup, A^1^b from A^3^ * | 35 | 13.3 | 54 | 32 |  | 14 | 28 |  | e-1b |
| N/A | 3 | 1.1 | - | - | - | - | - | - | - |
| **A^2^** | **Our study (n=263)** | | **Yamashita (n=170)** | | ***p* Value** | **Boyden (n=50)** | | ***p* Value** | **Fig.** |
|  | **No.** | **%** | **No.** | **%** |  | **No.** | **%** |  |  |
| A^2^a from A. rec, A^2^b from A. asc | 122 | 46.4 | 91 | 53.8 | 0.14 | 21 | 41 | 0.56 | e-2a |
| A^2^a + A^2^b from A. asc | 75 | 28.5 | 51 | 30.1 | 0.74 | NR | - | - | e-2b |
| A^2^a + A^2^b from A. rec | 39 | 14.8 | 25 | 14.9 | 0.97 | 29 | 58 | < 0.001 | e-2c |
| A^2^b (or A^2^b + A^2^a) from A^3^ ** | 11 | 4.2 | NR | - | - | NR | - | - | e-2d |
| A^2^b from A^6^, A^2^a from A. rec | 7 | 2.7 | NR | - | - | NR | - | - | e-2e |
| A^2^a + A^2^b from A^6^ | 6 | 2.3 | NR | - | - | NR | - | - | e-2f |
| N/A | 3 | 1.1 | - | - | - | - | - | - | - |
| **A^3^** | **Our study (n=263)** | | **Yamashita (n=170)** | | ***p* Value** | **Boyden (n=50)** | | ***p* Value** | **Fig.** |
|  | **No.** | **%** | **No.** | **%** |  | **No.** | **%** |  |  |
| A^3^a + A^3^b from Tr. Sup | 180 | 68.5 | 103 | 60.5 | 0.94 | 35 | 70 | 0.82 | e-3a |
| A^3^b from Tr. sup, A^3^a from A. asc | 34 | 12.9 | 40 | 23.5 | 0.004 | 15 | 30 | 0.002 | e-3b |
| A^3^a + A^3^b from Tr. Inf | 20 | 7.6 | 5 | 3.0 | 0.04 | NR | - | - | e-3c |
| A^3^a from Tr. sup, A^3^b from Tr. inf | 20 | 7.6 | 11 | 6.5 | 0.65 | NR | - | - | e-3d |
| A^3^b from Tr. inf, A^3^a from A. asc | 5 | 1.9 | 11 | 6.5 | 0.01 | NR | - | - | e-3e |
| A^3^a from A^4+5^, A^3^b from Tr.sup | 1 | 0.4 | NR | - | - | NR | - | - | e-3f |
| N/A | 3 | 1.1 | - | - | - | - | - | - | - |

N/A = not available

NR ­­= the type was not referred.

* A^1^a branching directly from the Tr. sup, and A1b branching from A^3^ that bifurcated from the Tr. sup.

** This type was further divided into four subtypes. (1) A^2^b branching from A^3^ bifurcated Tr. sup, and A^2^a branching from A. asc; seven cases. (2) A^2^a and A^2^b branching from A^3^ bifurcated the Tr. sup; two cases. (3) A^2^b with A^3^ branching from Tr. inf, and A^2^a branching from A.rec; one case. (4) A^2^a and A^2^b with A^3^ branching from Tr. inf; one case.
