# Supplementary material for: An analysis of variations in the bronchovascular pattern of the right upper lobe using three-dimensional CT angiography and bronchography
Source: Gen Thorac Cardiovasc Surg. 2015 Feb 28;63(6):354–60. doi: 10.1007/s11748-015-0531-1 (PMC4454828; doi:10.1007/s11748-015-0531-1)
Supplement: Supplementary file 2 — Supplementary material 2 (PPTX 3675 kb) [file 11748_2015_531_MOESM2_ESM.pptx]

## Slide 1
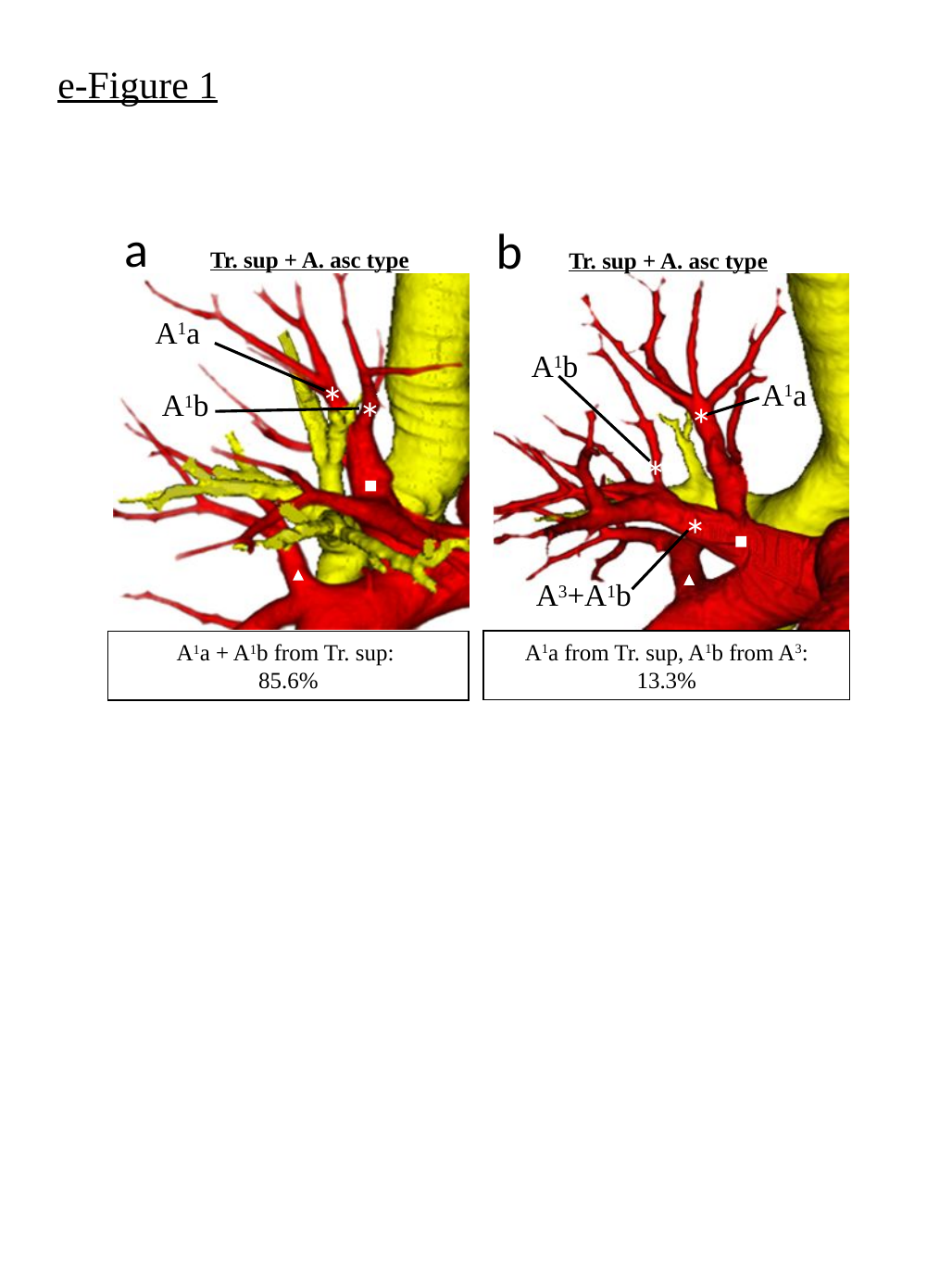

# e-Figure 1
a
b
A1b
A1a
*
*
*
A3+A1b
A1a from Tr. sup, A1b from A3: 13.3%
Tr. sup + A. asc type
Tr. sup + A. asc type
A1a
*
A1b
*
A1a + A1b from Tr. sup:
85.6%

## Slide 2
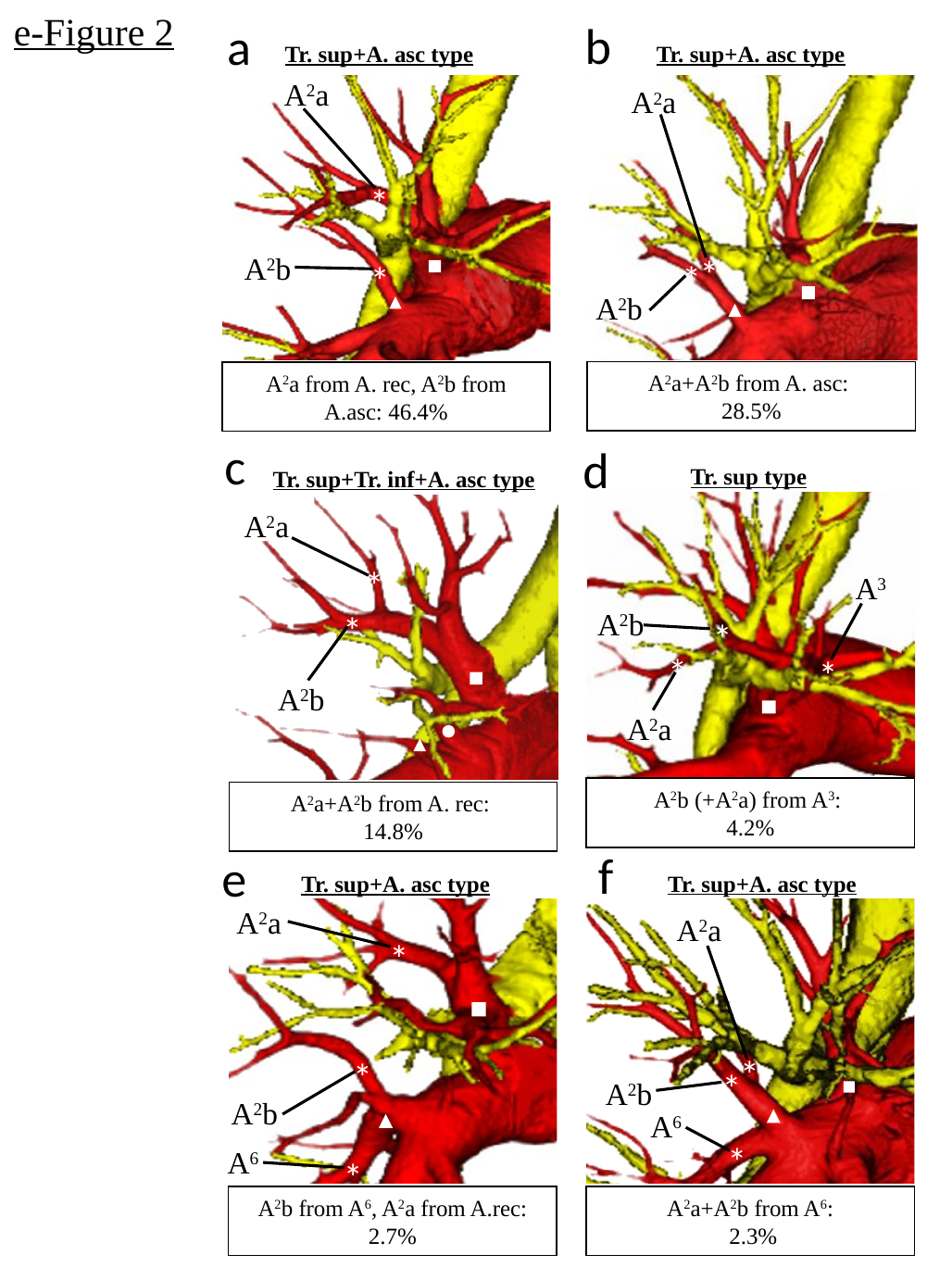

# e-Figure 2
b
Tr. sup+A. asc type
A2a
*
*
A2b
A2a+A2b from A. asc:
28.5%
a
Tr. sup+A. asc type
A2a
*
A2b
*
A2a from A. rec, A2b from A.asc: 46.4%
c
A2a
*
*
A2b
Tr. sup+Tr. inf+A. asc type
A2a+A2b from A. rec:
14.8%
d
Tr. sup type
A3
A2b
*
*
*
A2a
A2b (+A2a) from A3:
4.2%
f
e
Tr. sup+A. asc type
A2a
*
*
A2b
A6
*
A2b from A6, A2a from A.rec: 2.7%
Tr. sup+A. asc type
A2a
*
*
A2b
A6
*
A2a+A2b from A6:
 2.3%

## Slide 3
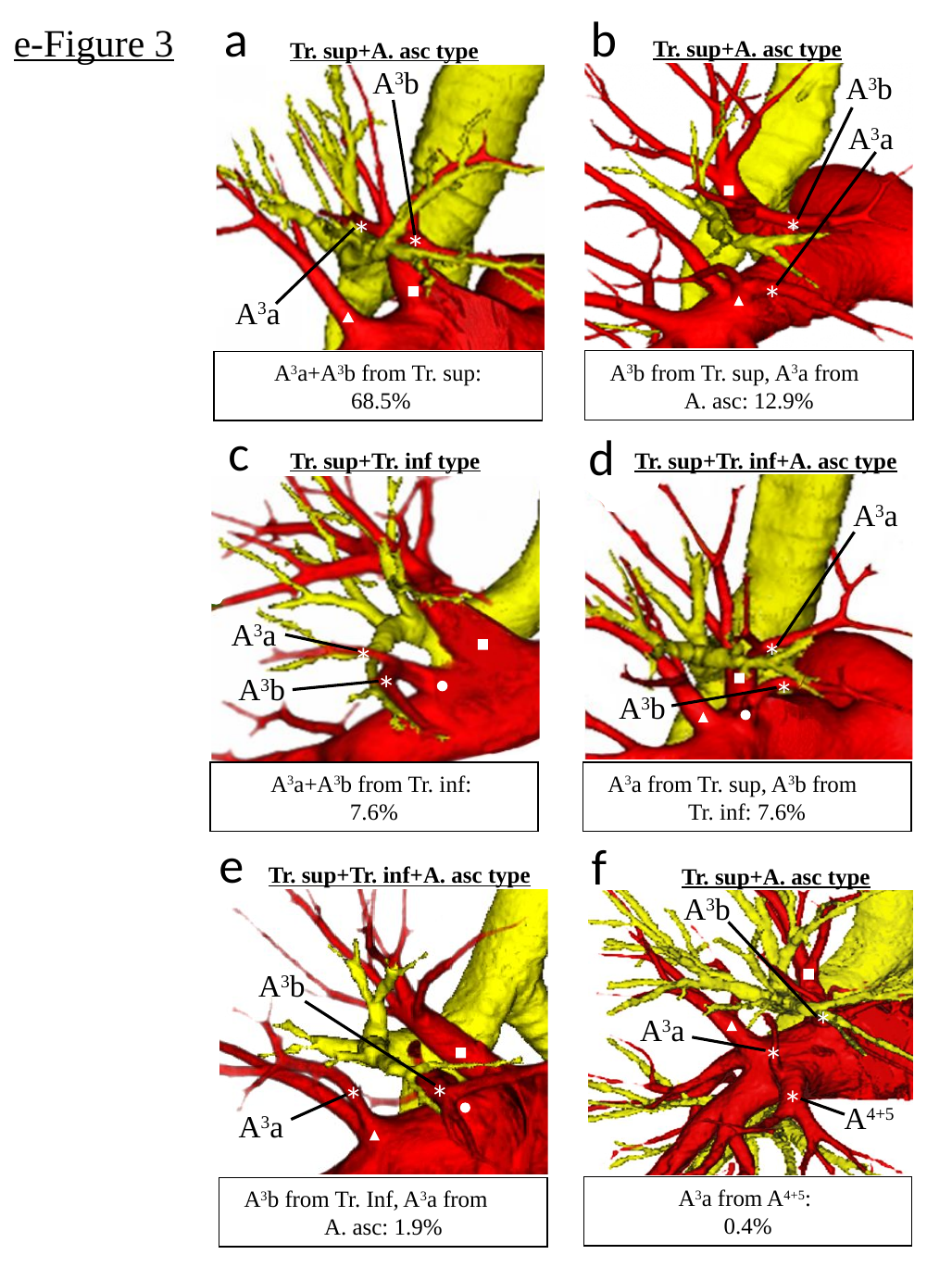

b
Tr. sup+A. asc type
A3b
A3a
*
*
A3b from Tr. sup, A3a from A. asc: 12.9%
a
Tr. sup+A. asc type
A3b
*
*
A3a
A3a+A3b from Tr. sup:
 68.5%
# e-Figure 3
c
Tr. sup+Tr. inf type
A3a
*
*
A3b
A3a+A3b from Tr. inf:
7.6%
d
Tr. sup+Tr. inf+A. asc type
A3a
*
*
A3b
A3a from Tr. sup, A3b from Tr. inf: 7.6%
e
Tr. sup+Tr. inf+A. asc type
A3b
*
*
A3a
A3b from Tr. Inf, A3a from A. asc: 1.9%
f
Tr. sup+A. asc type
A3b
*
A3a
*
*
A3a from A4+5:
0.4%
A4+5

## Slide 4
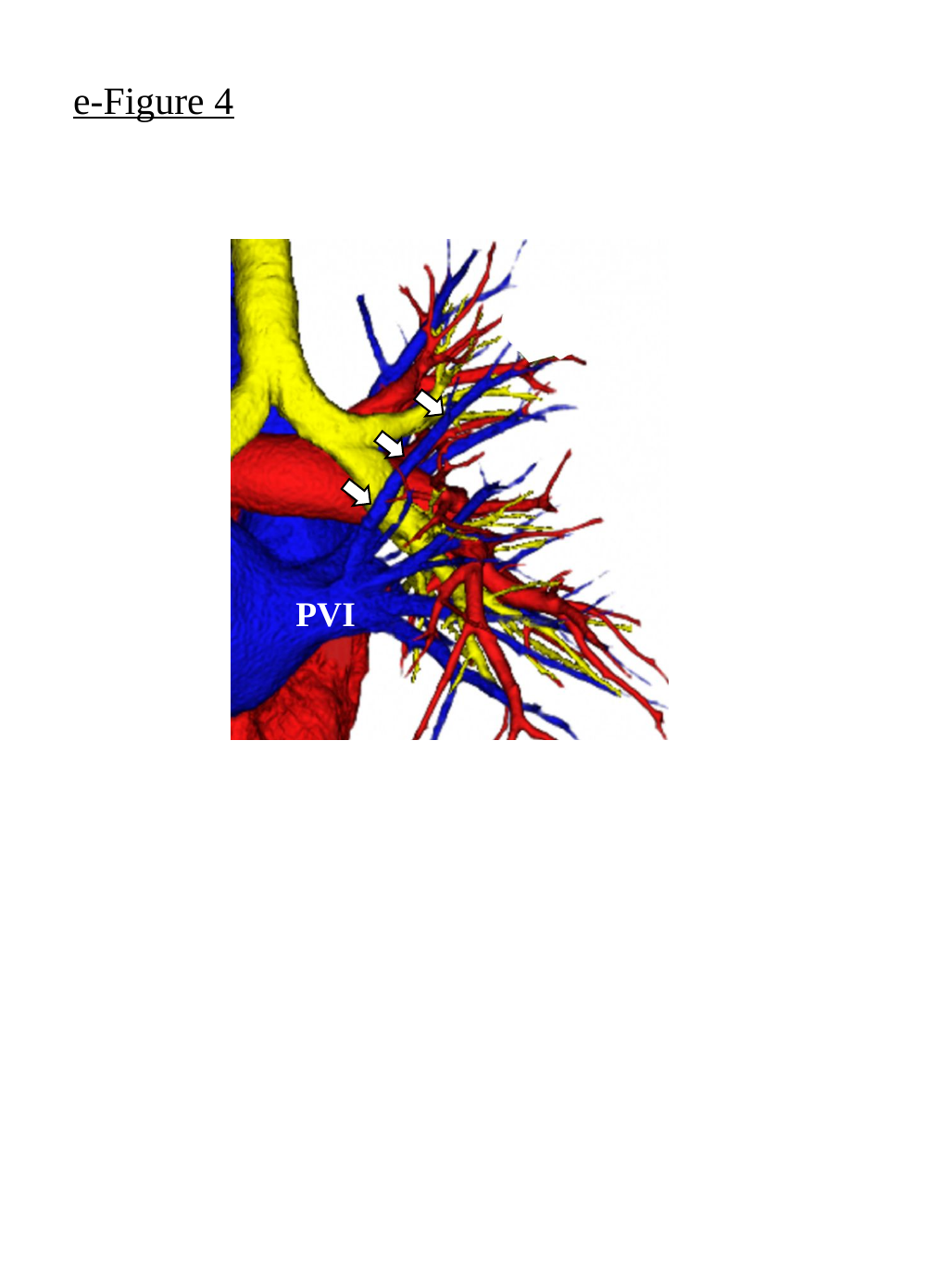

# e-Figure 4
PVI
